# Predictors of low back disability in chiropractic and physical therapy settings

**DOI:** 10.1186/s12998-020-00328-3

**Published:** 2020-08-12

**Authors:** M. John Petrozzi, Sidney M. Rubinstein, Paulo H. Ferreira, Andrew Leaver, Martin G. Mackey

**Affiliations:** 1 Faculty of Medicine and Health, University of Sydney, Rm S223, S Block, Sydney School of Health Sciences, Cumberland Campus NSW, Sydney, 2141 Australia; 2 Department of Health Sciences, Vrije Universiteit, Amsterdam, The Netherlands

**Keywords:** Chronic non-specific LBP, Predictors, Prognosis, Physical therapy, Chiropractic

## Abstract

**Background:**

Predicting ongoing disability for chronic non-specific low back pain (LBP) is important to avoid prolonged disability.

**Objective:**

Determine predictors of disability at 6 month follow-up in patients with LBP at medium risk of ongoing disability.

**Methods:**

Baseline data was collected from 108 patients with medium-risk chronic non-specific LBP (mean age 50.4 years, SD 13.6) from six private chiropractic and physiotherapy clinics in Australia who took part in a randomised control trial. All patients received a pragmatic course of multimodal physical treatments [e.g., manual therapy (spinal manipulation or mobilization and/or soft tissue massage)] combined with advice, education and exercise. Baseline prognostic variables included sociodemographic, physical and psychological characteristics. Primary outcome was disability (Roland Morris Disability) at 6 month follow-up. Multivariable linear regression analysis was conducted.

**Results:**

Variables remaining in the final multivariable model: lower work ability (β = − 1.05, 95% CI − 1.40 to − 0.70; p < 0.0001) and consultation with a medical specialist for back pain in the preceding 3 months (β = 3.35, 95% CI 1.14 to 5.55; p < 0.003), which significantly predicted higher disability at 6 months (unadjusted R ^2^ = 0.31). Those with a lower work ability (scale 1 to 10) and who had seen a medical specialist for their back pain were more likely to report greater LBP-related disability at 6 months.

**Conclusion:**

Patients with chronic LBP presenting to primary care with lower work ability and recent consultation with a medical specialist for LBP are more likely to have a worse prognosis; these are indicators to clinicians that standard conservative care may not adequately manage the patients’ needs.

## Introduction

Low back pain (LBP) is a significant health problem affecting one in ten people at any time worldwide. It has the highest global burden of disease for years lived with disability [1], and a lifetime recurrence of up to 85% [2]. Compared to other chronic health problems, LBP is the most common condition forcing people out of the workplace, and LBP-related psychosocial factors are predictive for poor outcomes [3]. Identifying factors or patient characteristics that predict the outcome of low back pain-related disability is important for informing the patient with prognostic advice about recovery, recurrence, appropriate treatment choice and referral to more appropriate care.

Previous research identified 36 prognostic factors associated with poor outcomes and disability in LBP cohorts across sociodemographic, physical, psychological, occupational and social domains [3–12]. This underscores the multidimensional nature of LBP and its prognosis [13]. Several systematic reviews [3–6] and high-quality studies [7–12] have consistently reported a range of factors predictive of poor outcomes and disability, among which are back pain intensity and duration, higher level of functional disability, older age, previous medical care for LBP, psychological or psychosocial stress, lower work ability, treatment-seeking behaviour and the patient’s satisfaction with their response to treatment, presence of compensation, poor relations with colleagues and heavy physical work demands.

Although physical and psychological prognostic factors have been identified in both acute and chronic LBP cohorts [8, 9, 14], these studies included a heterogeneous mix of LBP cohorts characterised by varying degrees of risk of disability (i.e., not stratified according to patient risk profiles). Targeting treatment to subgroups of patients based on prognostic characteristics and risk of disability is important for successfully delivering targeted treatments [15]. Furthermore, some LBP patients rated at medium risk of delayed recovery by the STarT Back screening tool (SBST) that do not respond to physical treatments as expected may continue to experience ongoing disability due to psychosocial factors not previously detected [16]. Previous research has not investigated prognostic factors for LBP disability in an homogenous stratified group, such as patients at medium risk of ongoing disability [7]. Those at medium risk are an important target population to study as these individuals present to primary care with little to no psychosocial issues, compared to those at high risk [17]. Therefore, identifying prognostic factors in this population at an early stage of management may lead to more accurate prognostic advice about recovery and recurrence, help direct appropriate treatment choices and enable early identification or referral to more appropriate care as needed.

The aim of this study was to examine the clinical and socio-demographic baseline factors that predict greater disability in people attending physiotherapy and chiropractic practices with chronic LBP at medium risk of ongoing disability.

## Methods

### Study design

A secondary analysis of a multi-centre cohort study was conducted in patients with chronic non-specific LBP at medium risk of ongoing disability who participated in the ‘Mind Your Back’ randomised controlled trial (RCT) [18, 19]. The trial protocol and outcomes have been reported elsewhere [18, 19]. The Strengthening the Reporting of Observational Studies in Epidemiology (STROBE) checklist was used for transparent reporting of this study [20].

### Participants

Trial participants attended six chiropractic and physiotherapy clinics in metropolitan New South Wales and Victoria, Australia. Recruitment commenced on March 30, 2015 and ended June 2017. Participants analysed in this present study provided baseline and follow-up data at 6 months. Clinicians were recruited through advertisements in professional journals and by referral. All participants received standard care (multimodal physical treatment), with half of the cohort randomised to an intervention arm to access an online psychological program (MoodGYM) in addition to standard care. As no between-group differences in primary outcomes were found at any follow-up time point [18, 19], a homogenous cohort of participants was subsequently assembled for this study.

Inclusion criteria were as follows: > 18 years of age; a history of non-specific LBP of greater than three months’ duration; did not receive manual therapy for their condition in the previous three months; and at medium risk of disability according to the STarT Back Screening Tool [17]. Exclusion criteria were as follows: a diagnosis of serious spinal pathology (fracture, malignancy, infection, inflammatory disorders, canal stenosis or cauda equina syndrome, spinal cord injury); spinal nerve compromise (determined by the presence of two or more corresponding neurological signs such as dermatomal paraesthesia, myotome weakness, diminished or absent deep tendon reflexes); spinal surgery in the previous 12 months, pregnancy, and current worker’s compensation claim related to their back condition. Also excluded were participants who were unable to independently complete English language questionnaires, or unable to independently use a computer. Participants entered baseline data directly into an online survey collection database (Survey Monkey©) before any treatment or consultation with a follow-up at 6 months.

**Table 1:** Participant characteristics at baseline (values are mean ± SD for continuous variables and N (%) for categorical variables)

Characteristic	Value (*n* = 108)
***Sociodemographic***	
Age (years)^a^	50.4 (13.6)
Gender	
-Male	54 (50%)
-Female	54 (50%)
BMI	26.8 (4.5)
Diagnostic tests for back pain in past 3 months^a^	51 (47.2%)
Consultation with medical specialist for back pain in the last 3 months^a^	14 (13.0%)
Analgesics for back pain^a^	54 (50%)
***Physical profile***	
*LBP intensity, frequency and duration*^a^	
LBP intensity PNRS (0–10) ^a^	5.0 (1.9)
Always present, level of pain varies	68 (63.0%)
Often present, with pain-free periods < 6 h	24 (22.2%)
Other	16 (14.8%)
*Functional Status*	
RMQ (0–24)^a^	9.9 (4.4)
PSFS (0–10)	4.2 (1.4)
***Psychological status***	
WAS (0–10)^a^	5.7 (2.1)
PSEQ (0–60)^a^	44.5 (12.3)
PCS Total (0–52)^a^	20.5 (11.9)
DASS21 Total (0–63)^a^	15.9 (11.4)

NB: BMI (Body Mass Index); PNRS (Pain Numeric Rating Scale); RMQ (Roland Morris Disability Questionnaire); PSFS (Patient Specific Functional Scale); WAS (Work Ability Score); PSEQ (Patient Self-Efficacy Questionnaire); PCS (Pain Catastrophising Scale); DASS21 (Depression Anxiety Stress 21-item Scale). Selected potential putative predictor variables were based on previously investigated factors reported in the literature as prognostic for disability and based on significance of univariate regression analysis, the selected putative predictor variables (^a^) were included in the multivariable models at 6 months

### Putative predictor variables

From a large group of available baseline data, 15 putative predictor variables were selected a priori based on: 1) previous evidence in the literature of a strong association with pain and disability in LBP cohorts [3–6]; and 2) a p < 0.2 association with the dependent variable (disability) from univariable analysis. Predictor variables were categorised into three broad domains (Table 1): sociodemographic (i.e., age, gender, BMI, diagnostic testing in the previous three months and consultation with a medical specialist within the previous three months); physical profile (i.e., LBP frequency and intensity, general functional capacity, specific functional capacity, response to treatment and analgesic use); and psychological profile (i.e., pain self-efficacy, pain catastrophising, depression/ anxiety/ stress and work ability belief).

*Categorical predictor* variables:

**Table:** 

• Gender (M/F) • Visited a medical specialist for back pain in last three months (Y/N) • Radiographic diagnostic imaging for back pain in last three months (Y/N) • Analgesics use (Y/N) • LBP frequency was categorised into 5 sub-groups according to the frequency and intensity of LBP experienced immediately prior to recruitment: always present (always the same intensity), always present (but level of pain varies), often present (pain-free periods lasting less than 6 h), occasionally present (pain occurs once to several times per day, lasting up to an hour), and rarely present (pain occurs every few days or weeks)	

*Continuous predictor* variables:

**Table:** 

• Age (years) • BMI (kg.^m-2^) • Usual LBP intensity (in the week prior to starting the trial) measured by the Pain Numeric Rating Scale (PNRS) (0–10 point scale) [21] • Self-reported disability measured by the Roland Morris Disability Questionnaire (RMQ) (0–24 point scale) [22] • Work ability (now compared to lifetime best) measured by the single item Work Ability Score (WAS) (1–10 point scale). The Work Ability Score is strongly associated with the overall Work Ability Index score [23] • Specific functional capacity was assessed with the Patient Specific Functional Scale (PSFS) (0–10 point scale) [24] • Self-efficacy measured by the Pain Self-Efficacy Questionnaire (PSEQ) (0–10 point scale) [25] • Pain catastrophising measured by the Pain Catastrophising Scale (PCS) (0–52 point scale) [26, 27] • Depression, anxiety and stress measured by the overall Depression Anxiety Stress Scale-21 (DASS21), (0–21 point scale) [28] • Response to treatment was assessed as the difference in pain numeric rating scale (PNRS) between clinical visit 1 to visit 3 (0–10 point scale). Pain intensity was measured immediately before the first physical treatment session (baseline) and immediately before the third physical treatment session. Previous research has reported that improvement in LBP scores early in treatment of LBP can predict long-term disability [29–33]	

#### Outcome variable

The clinical outcome variable was self-reported LBP-related disability, measured by the RMQ (0–24 point scale) at 6 months. RMQ was selected as the primary outcome measure for several reasons. First, it is a reliable measurement for inferring LBP-related disability and is sensitive to longitudinal change over time for a patient with chronic LBP [34, 35]. Second, monitoring disability in practice is clinically important for practitioner and patient as it is often reported as a measure of LBP progression [36]. Third, RMQ was selected over pain measures (e.g., PNRS) as patients with chronic LBP report reduced physical functioning because of their pain; this is supported by the literature, which recommends that assessment of function should be an integral part of pain assessment [37, 38]. RMQ at 6 months was selected over the 8 week and 12 month time periods due to several factors: the participants had lived with chronic LBP for 3–5 years, and clinicians may consider an 8 week period too short to expect improvements in disability for this cohort; the sample size reduced by 9% at 12 months; and the larger sample of participants at 6 months enabled a rigorous multiple regression analysis, allowing a meaningful research question to be answered for practitioners.

### Statistical methods

Means and standard deviations (SD) are presented for continuous baseline variables, and frequency distributions are presented for categorical variables (Table 1). All participant data were included at baseline (n = 108) as there were no missing data. Participants with missing data at 6 months (4.6%, n = 5) were excluded from the analysis leaving a total sample size of n = 103. To ensure the final 6 month multivariable regression model did not contain spuriously associated variables, a cross checking process was carried out on 8 week data (n = 106; drop-out 1.9%, n = 2) and 12 month data (n = 98; drop-out 9.3%, n = 10). The sample size required to support a multiple regression model depends on both the R value of the model and the number of variables that are included [39, 40]. An approximate sample size of 105 participants with an R^2^ of 0.4 is required for a study such as ours with 11 independent predictors [39]. Therefore, the missing data at 6 months was within acceptable limits, leaving a sample size of n = 103 for multivariable regression modelling. The 8 week data (n = 106) was also within acceptable limits, while the 12 month sample size (n = 98) was borderline. This meant that a process of imputing missing data was not required, which avoided falsely increasing the power of the study and potentially biasing the results.

#### Regression analysis

Univariate regression was performed between each predictor variable and the dependent variable at 6 months follow-up. The same procedure was used to analyse predictor and dependent variables at post-treatment (8 weeks) and at the 12 month follow-up to validate that the combination of variables in the final model was theoretically and clinically sound. All putative predictor variables with p < 0.1 were provisionally selected for inclusion in the multivariable model; this is a widely used method for testing variables before multivariable modelling [41] (Table 1).

All predictor variables with p < 0.1 significance level were assessed for multi-collinearity in order to identify any highly associated variables and exclude them from the analysis before running the multivariable model. Continuous and categorical predictor variables were analysed for multi-collinearity separately. For continuous predictor variables, multi-collinearity was indicated if Pearson’s correlation coefficients were > 0.7. Categorical predictor variables were also explored for multi-collinearity by conducting chi-square tests. All categorical variables were included in the multivariable model as no predictor variables were strongly related to each other.

Lastly, the full model containing all the independent variables was reduced using multivariable regression. This reduction process involved removing the least significant variable (p > 0.05) from the full model in a backward selection process while maintaining a strong overall F-test in order to maintain the strongest model at each step of the analysis [42]. This process was sequentially repeated until only the statistically significant variables were retained, resulting in the strongest predictive model. The goodness of fit of the final model was assessed by inspecting plots of the residual values against predicted values. These plots are useful in assessing the normality of residuals as well as homoscedasticity. As an additional check of multi-collinearity, a variance inflation factor of 1.2 and condition index of 6.2 confirmed that the variables in the final model were independent of each other.

Overall, there were no indications to suggest that the main assumptions of multivariable regression analysis were not supported [40]. That is, all important explanatory variables were included in the model, the variables were independent of each other and collected in a period when the relationship between the variables remained constant, the explanatory variables were independent of each other, the relationship between each explanatory variable and the outcome was linear, the residuals were normally distributed and there were no influential outliers. The IBM Statistical Package for Social Science (SPSS) (version 24) was used and all p-values are two-sided. We used a p < 0.05 level of statistical significance.

## Results

### Participants

One hundred and eight (108) participants (mean age 50.4 years, SD 13.6) with chronic LBP at medium risk of ongoing disability took part in the study. Details of participant screening and flow throughout the trial have been reported elsewhere [19]. Overall, 361 volunteers were screened for eligibility, of which 253 did not fulfil the inclusion criteria: 168 (66.5%) were classified as at either high-risk or low-risk of ongoing disability according to the STarT Back tool, and 80 (31.6%) did not meet other inclusion criteria, leaving 108 participants who were enrolled. There were no significant participant characteristic differences between the two groups that received the intervention or the control at baseline or follow-up. Therefore, both groups of participants were combined into one single homogenous cohort for the purposes of the present study.

In general, participants were middle-aged, slightly above normal weight, had a moderate level of work ability, with a moderate level of back pain, and low disability. They also demonstrated high self-efficacy and had normal to mild levels of psychological distress (Table 1). Approximately, two-thirds of participants had constant back pain for more than five years. In total, 40% were in full-time employment but approximately 50% stated that the number of hours worked each week was affected by pain. Further, the type of employment of 62% of participants was influenced by the experience and expectations of back pain. Forty-four percent did not attribute a specific cause to their back pain. The two most common comorbidities by participants were depression and anxiety (24.1%), and osteoarthritis (18.5%). Moreover, 47.2% had diagnostic imaging for back pain in the previous 3 months (x-ray or advanced imaging), 50% were medicated with analgesics (over the counter or prescribed) and 13% had consulted a medical specialist for back pain in the previous 3 months.

### Outcome data

#### Univariate analyses

Results of the univariate regression analysis of putative predictor variables satisfied the assumptions for using regression [39]. Associations showing p values < 0.1 between putative predictor variables and disability at 6 months were considered statistically significant. Gender, BMI and patient specific function scale were not statically associated with disability at 6 months and were not included in the full multivariable analysis model. Age, frequency of LBP episodes, visited a medical specialist for back pain in the previous 3 months, diagnostic tests for back pain in the previous 3 months, LBP intensity, disability, work ability, self-efficacy, catastrophising, combined depression/ anxiety/ stress scale, analgesics and change in pain scores between visits 1 to 3 were all significantly associated with disability at 6 months.

Results of multi-collinearity testing found that each of the putative predictor variables was independent of the others (Pearson’s p ≤ 0.7); however, the relationship between work ability and self-efficacy was borderline with a Pearson’s p = 0.701, and therefore both of these predictor variables were included in the multivariable analysis. Eleven predictor variables were retained for inclusion in the multivariable analysis after multi-collinearity testing.

#### Multivariable analyses

The final multivariable model retained two predictor variables associated with disability at 6 months that explained 31% (unadjusted R^2^ = 0.31) of the variance (Table 2). The two variables that predicted disability at 6 months were work ability (coef. − 1.05, 95% CI − 1.40 to − 0.70; p < 0.0001), and consultation with a medical specialist for back pain within the previous 3 months (coef. 3.35, 95% CI 1.14 to 5.55; p < 0.003). Both of these variables were also retained in final multivariable models at the 8 week and 12 month follow-up, and explained 35 and 25% respectively of the variability in disability at those time points, providing validity of their inclusion in the final multivariable model.

**Table 2:** Final multivariable model at 6 months (*n* = 103)

	Coef.	Std. Err.	t	*p*-value	95% CI
Consultation with medical specialist for back pain in the last 3 months	3.35	1.11	3.01	0.003	1.14 to 5.55
Work ability	−1.05	0.18	−5.91	< 0.001	−1.40 to −0.70
Constant	10.67	1.09	9.81	< 0.001	8.51 to 12.83
R-squared = 0.31					
Adjusted R-squared = 0.30					
Condition Index 6.2					

Although our sample size complies with recommendations for regression analysis [39], it is at the lower acceptable limit. Therefore, to cross-check the precision of the final multivariable regression model and to address potential issues with sample size, a multivariable regression analysis was performed on smaller groups of variables related to sociodemographic, physical and psychological domains. That is, six sociodemographic variables (age, BMI, gender, diagnostic testing in last 3 months, consultation with medical specialist for back pain in the last 3 months, analgesics), four physical profiles (LBP frequency, LBP intensity, RMQ, PSFS) and four psychological variables (PSEQ, PCS, DASS21, WAS) were applied to multivariable regression analysis while controlling for baseline RMQ and treatment group. From these domain analyses the variables from the strongest regression models (highest R^2^ value and lowest condition index) were selected for a further multivariable regression analysis. The resultant five independent variables were as follows: diagnostic testing in last 3 months, consultation with medical specialist for back pain in the last 3 months, analgesics, LBP intensity and work ability score. These variables were further analysed with multivariable regression; two variables – consultation with a medical specialist for back pain in the last 3 months, and work ability – gave R^2^ = 0.31, adjusted R^2^ = 0.30 and condition index = 6.2, confirming the results found in our initial analysis using the methodology outlined in the methods section. As such, this cross-checking confirmed that the strongest predictive model is presented in this study.

## Discussion

The primary aim of this secondary analysis was to examine the clinical and socio-demographic predictors of increased disability in people with chronic LBP in primary care at 6 months follow-up. Multivariable regression analyses revealed that lower work ability and consultation with a medical specialist for back pain within the last 3 months explained 31% of increased future disability at 6 months.

These findings are not supported by the consensus of evidence in the literature, which in contrast have reported that high disability, high pain intensity, high psychological distress and poor general health predicted greater ongoing chronic LBP disability [9–11]. It is possible these predictors were not found in the final multivariable model in our study because we selected a homogenous cohort of people at medium risk of ongoing disability. Earlier studies included heterogeneous cohorts of people with chronic LBP, not stratified for risk of disability [9–11, 43]. Second, the clinical profile of our study participants differed to those in previous studies. Our participants were characterised by moderate pain, low disability and high self-efficacy at baseline. Previous studies included participants with high pain and high disability [9, 44–46]. Researchers have previously highlighted the importance of research to study chronic LBP according to varying levels of risk stratification [15, 17]. Therefore, there is evidence that supports our final multivariable model.

### Work ability

Although the work ability construct is not an outcome routinely measured in LBP studies, there is some evidence that supports its inclusion, as well as our findings that work ability was a predictor for disability. For example, low work ability has been shown to be a strong risk factor for future work-related disability [47]. High work ability at baseline in a cohort of people attending physiotherapy for LBP was associated with less disability [48]. Research also suggests that it is important to screen chronic non-specific LBP patients for perceived work ability during health consultations because low work ability is associated with poorer general health and lower self-efficacy beliefs (R^2^ 0.42) [12, 49]. This is important as low self-efficacy beliefs are associated with higher disability [50–52]. Therefore, it is argued that the concept of work ability [23] and its measurement in a chronic LBP population is an important and valid means of measuring a person’s perceived ability to participate in work. Furthermore, work ability is a valid predictor of future disability in people with medium-risk chronic LBP, as evidenced by the present study.

### Consultation with a medical specialist for back pain within the last 3 months

Although the majority of people experiencing an episode of LBP do not routinely seek care [53–55], those with chronic LBP are more likely to seek care from a medical or allied health professional due to the negative influence of pain and disability on their health status [54–56]. People with LBP (18%) experience one or more recurrences over 1 year and seek medical care [57]. While seeking care is more likely in those with high levels of LBP and disability, those with low-intensity LBP and disability also seek care [58]. These characteristics are similar to our participants, who had moderate levels of back pain and low levels of disability at baseline. People with chronic LBP also utilise more medical services compared to the average population [56], with greater use of pain-related medications and increased medical care utilisation [58] accounting for part of the total economic burden of chronic LBP in developed countries (average 1.2–2.3% GDP [59, 60]. Those that seek care from allied health practitioners (e.g., physiotherapists) (4.5%) for LBP often report they had also sought care from a medical professional (GP or medical specialist) at least once over a two-month period [61] and those with chronic LBP sought medical and allied care more frequently [61].

Participants in our study reported normal to mild levels of psychological distress. The normative data in the literature for the DASS-21 are as follows: scores for the depression scale for ‘Normal’ are 0–4, ‘Mild’ are 5–6; Anxiety scale for ‘Normal’ are 0–3, ‘Mild’ are 4–5; Stress scale for ‘Normal’ are 0–7, ‘Mild’ are 8–9 [28]. Previous studies have noted that people with chronic LBP who had high-intensity LBP and depression reported the highest rates of care-seeking from medical and allied health professionals compared to others with low depression [62]. Studies have also noted that predictors for seeking care over a six-month period, for people with chronic LBP, are psychosocial factors (pain-related stress, anxiety and depression) [63]. Qualitative studies further highlight that people with chronic LBP repeatedly seek care from primary and specialist medical professionals throughout their lives as they are frustrated by the ongoing nature of their condition, disability, and lack of curative treatments offered by their practitioner [64, 65]. Therefore, people who have sought medical care and are seeking further care from a chiropractor or physiotherapist, such as those in our study, tend to repeatedly seek care without experiencing long term improvements in disability. The above evidence supports our findings that prior consultation with a medical specialist is predictive of long-term disability in people with chronic LBP.

## Limitations and strengths

There are several limitations to this study. First and perhaps most importantly, the final model explained only 31% of the disability. Therefore, many other variables that we did not examine could potentially better explain the outcome of the participants; thus, the results may potentially be of limited clinical utility. Secondly, there is a possibility that a heterogeneous group of patients may have been recruited to the study despite the stringent participant inclusion criteria. That is, participants were not only recruited from internal methods of advertising at the time of seeking care (e.g., notice board and brochures in GP and allied health centres), but also via external methods (e.g., newspaper editorials and university trial recruitment website); therefore, this may have introduced participants who may not otherwise have sought care for their condition. Third, the approach of pooling and analysing participants from a randomised trial into one single cohort to identify associations between measures may be less representative of the broader clinical population due to the focused eligibility criteria necessary for the randomised trial. Fourth, the sample size was borderline sufficient for supporting 11 independent variables; however, reanalysis with smaller numbers of variables by domain confirmed the final multivariable model.

A unique aspect of this study is the focus on the ‘medium-risk of ongoing disability’ group. Previous research has examined the predictors of disability in acute [5, 7, 8, 66, 67] and chronic LBP cohorts [7, 9, 68–70]; however, those studies focused on a heterogeneous LBP cohort. To date, no previous study has investigated predictors of future disability in people attending primary care for chronic LBP at medium-risk of disability.

The results of this study can be pooled with other studies of stratified cohorts and used to inform future clinical practice guidelines for the treatment of chronic non-specific LBP for medium-risk cohorts [71–74]. This is important, as researchers have suggested that clinical practice guidelines need to specifically recommend that practitioners provide care according to a patient’s risk profile (e.g., low, medium or high risk) [15]. The results of this study are generalisable to chronic LBP populations at medium risk of ongoing disability seeking care from a chiropractor or physical therapist.

## Interpretation

Previous literature has highlighted the importance of identifying simple prediction rules appropriate for busy clinical settings [75, 76]. While acknowledging that the predictive ability of this model was quite low, chiropractors and physical therapists could potentially use an easy and quick method for evaluating the risk of long-term disability in their patients with chronic LBP at medium risk of disability by asking two simple questions: ‘Have you seen a specialist for back pain within the last 3 months? (y/n)’, and ‘How do you rate your work ability now compared to your lifetime best on a scale of 1–10 (1 is worst / 10 is best)’. The results of this analysis suggest that it would be beneficial for patients with chronic LBP at medium-risk of ongoing disability to complete the work ability index at first consultation and be asked if they have seen a medical specialist for LBP in the last three months. Such early screening could potentially aid the identification of patients requiring further investigation of health or work-related contributors to perceived poor work ability, as well as patients who may experience future disability based on clinical and demographic predictors. Furthermore, future research is required to understand how these prognostic factors can assist clinical decision making.

## Conclusion

Patients presenting with lower work ability and recent consultation with a medical specialist for LBP are likely to experience higher levels of disability at 6 months. Identification of these clinically relevant predictors at the first visit may indicate to the practitioner that standard conservative treatments may not adequately address the patients’ impairments, with consideration of alternate management strategies to avoid prolonged disability.

## Data Availability

The datasets generated and/or analysed during the current study are not publicly available, as secondary analysis is currently being undertaken with the intention of being published but are available from the corresponding author on reasonable request.
